# Comparison of Thoracic Radiography and Computed Tomography in Calves with Naturally Occurring Respiratory Disease

**DOI:** 10.3389/fvets.2017.00101

**Published:** 2017-07-06

**Authors:** Jennifer Fowler, Susanne M. Stieger-Vanegas, Jorge A. Vanegas, Gerd Bobe, Keith P. Poulsen

**Affiliations:** ^1^Department of Clinical Sciences, College of Veterinary Medicine, Oregon State University, Corvallis, OR, United States; ^2^Department of Animal and Rangeland Sciences, College of Agricultural Sciences, Oregon State University, Corvallis, OR, United States; ^3^Linus Pauling Institute, Oregon State University, Corvallis, OR, United States; ^4^Wisconsin Veterinary Diagnostic Laboratory, Department of Medical Sciences, School of Veterinary Medicine, University of Wisconsin, Madison, WI, United States

**Keywords:** computed radiography, multi-detector computed tomography, calf, naturally occurring respiratory disease, pneumonia

## Abstract

**Objective:**

To evaluate the severity and extent of lung disease using thoracic computed radiography (CR) compared to contrast-enhanced multi-detector computed tomography (MDCT) of the thorax in calves with naturally occurring respiratory disease and to evaluate the feasibility and safety of performing contrast-enhanced thoracic multi-detector MDCT examinations in sedated calves. Furthermore, to evaluate if combining CR or MDCT with respiratory scoring factors will improve prediction of the chronicity of pulmonary disease in calves.

**Animals:**

Thirty Jersey heifer calves ranging in age between 25 and 89 days with naturally occurring respiratory disease.

**Procedures:**

All calves were evaluated *via* thoracic CR and contrast-enhanced MDCT. All calves were euthanized immediately following thoracic MDCT and submitted for necropsy. Imaging and histopathology results were compared with each other.

**Results:**

Thoracic MDCT was superior for evaluation of pneumonia in calves due to the lack of summation in all areas of the lungs. Intravenously administered sedation provided an adequate plane of sedation for acquiring MDCT images of diagnostic quality, without the need for re-scanning. A diagnosis of pneumonia was made with equal rate on both thoracic CR and MDCT. Although mild differences in classification of lung pattern and extent of lung disease were seen when comparing an experienced and a less experienced evaluator, the overall differences were not statistically significant. The best intra- and inter-observer agreement was noted when evaluating the cranioventral aspects of the lungs in either modality. Clinical respiratory scoring is inadequate for diagnosing chronicity of pneumonia in calves with naturally occurring pneumonia.

**Conclusion and clinical importance:**

Both imaging modalities allowed diagnosis of pneumonia in calves. The cranial ventral aspects of the lungs were most commonly affected. Thoracic CR and MDCT provided similar diagnostic effectiveness in diagnosing pneumonia. However, MDCT provided better assessment of subtle details, which may be otherwise obscured due to summation artifact.

## Introduction

Respiratory disease in cattle is the most prevalent cause of morbidity and mortality in beef feedlots, and the most common cause of morbidity in weaned dairy calves (1). Early diagnosis of disease is essential to reduce the risk of disease propagation and to decrease the financial costs from treatment and loss of animals. Furthermore, early diagnosis promotes good quality of life for the patient, improves response to treatment, and decreases the likelihood of prolonged morbidity following juvenile pneumonias (2, 3).

Calves with respiratory disease often present with fever, cough, and tachypnea. Auscultation is commonly used to further evaluate the lungs. However, several of these clinical findings have been proven unreliable to diagnose pneumonia in calves. As such, diagnosis of bovine respiratory disease remains a diagnostic challenge.

Thoracic radiography is the most commonly used test of choice to evaluate for pneumonia in human and veterinary patients. Radiography of the thorax of large animals can be limited to having only lateral projections of the thorax available for interpretation and by inadequate penetration due to the size of large animals and equipment limitations; however, its practicality including its use in field conditions, low cost, and widespread availability make it one of the most common used techniques to evaluate the thorax in large animals with respiratory disease (4–6). Computed tomography (CT) of the thorax is considered the modality of choice to evaluate for the presence of lung disease in human patients and is increasingly used in veterinary patients (7). Thoracic CT has proven to be helpful for evaluating the extent of pulmonary disease and provides better anatomic detail due to a lack of superimposition of anatomical structures (8). The use of CT in calves is often limited by the cost and lack of availability of a CT scanner close to a calf rearing operation. Furthermore, CT examinations of the thorax are commonly performed under general anesthesia with endotracheal intubation to allow using breath-hold techniques, which decrease the risk of respiratory motion (9, 10). However, calves with severe respiratory impairment due to pneumonia often have an increased anesthetic risk. Although CT scanning protocols have been described for awake and sedated patients including for normal cats and cats with upper airway obstruction and intrathoracic disease, dogs with acute abdominal signs, and dogs with traumatic pelvic fractures, as well as healthy sedated foals neither has been evaluated in calves to date (11–15).

Our objective was to compare the severity and extent of lung disease using computed radiography (CR) compared to contrast-enhanced multi-detector CT (MDCT) of the thorax and further determine the feasibility and safety of performing thoracic MDCT examinations in sedated calves with naturally occurring respiratory disease. We hypothesized that thoracic CR would be sufficient to diagnose lung disease in calves; but that MDCT in sedated calves could be safely performed and would provide more information about the extent of lung involvement. We further wanted to evaluate if combining CR or MDCT with respiratory scoring factors will improve prediction of the chronicity of pulmonary disease in calves.

## Materials and Methods

### Study Population

Thirty privately owned pre-weaned Jersey heifer calves with naturally occurring respiratory disease ranging in age between 25 and 89 days (50.5 ± 18.8 days) were included in the study. The calves with respiratory disease were identified by farm workers dedicated to calf health monitoring and treatment using a clinical respiratory disease scoring system (16). On the farm, the calves were grouped based on respiratory disease duration into acute disease (within 24 h of first signs of clinical pulmonary disease, group 1) and chronic disease. Calves with chronic pulmonary disease had received antibiotic treatment at the onset of clinical pulmonary disease but continued to exhibit clinical signs of pneumonia. Chronic disease calves were subdivided into two groups: group 2 included calves which had received one antibiotic treatment 1 week prior (short-term chronic disease), and group 3 included calves which had received antibiotic treatments 2 weeks and 1 week prior (long-term chronic disease).

To study the effect of naturally occurring respiratory disease duration on imaging findings out of each group (acute, short-term, and long-term chronic), ten calves were randomly selected and transported to the Lois Bates Acheson Veterinary Teaching Hospital at Oregon State University for the imaging studies. Calves were housed with free choice of water, calf starter, and were fed milk replacer twice daily until the imaging studies were performed. All imaging studies were performed within 24–48-h post respiratory scoring on the farm. The study was approved by the Oregon State University Institutional Animal Care and Use Committee.

### Thoracic Computed Radiography

Standing left to right lateral computed radiography (FCR ClearView CS IIP and Type C IP, Fujifilm Co., Tokyo, Japan) images of the thorax were obtained using 85kVp and 20–32 mAs at 630 mA. If calves were smaller in size, the exposure was adjusted by reducing the mAs to 20 and keeping the mA constant at 630 to ensure optimal image quality. One or up to three radiographs of the thorax were obtained to ensure that all aspects of the lungs were included in the study. All diagnostic images were sent to a designated image storage server for off-line analysis.

### Multi-Detector Computed Tomography

All thoracic CT studies were performed under sedation using a 64-row MDCT scanner (Toshiba Aquilion 64 CT, Toshiba America Medical Systems Inc., Tustin, CA, USA). Prior to the MDCT study, a jugular vein catheter was aseptically placed in each calf. Calves were sedated with a combination of 0.1 mg/kg butorphanol (Torbugesic^®^, Pfizer, New York, NY, USA), 0.3 mg/kg ketamine (Ketaset^®^, Pfizer/Boehringer, St. Joseph, MO, USA), and 0.4 mg/kg xylazine (Anased^®^, Lloyd Laboratories, Shenandoah, IA, USA) for the MDCT study. All thoracic MDCT scans were performed with the calves positioned in sternal recumbency. A non-contrast enhanced thoracic MDCT scan followed by a contrast-enhanced MDCT scan was performed in each calf from approximately 10 cm cranial to the thoracic inlet to the mid-level of the left kidney using the following scan parameters: 0.5-mm collimation, 0.5-mm reconstruction interval, 1 s tube rotation time, 120 kV, 400 mA, a pitch factor of 0.828, and 0-degree tilt. The contrast-enhanced MDCT scan was performed 60 s after the start of an intravenous iodinated contrast agent (Isovue 300, Bracco Diagnostics Inc., Princeton, NJ, USA) injection at 1 mL/kg using a power injector (Empower CTA, Bracco Diagnostics Inc., Princeton, NJ, USA) at a flow rate of 3 mL/s. The thin collimated MDCT isovolumetric data were used to create transverse, sagittal, and dorsal reconstructed images of the thorax with 3-mm slice thickness. A bone, soft tissue, and lung algorithm was used to create bone, soft tissue, and lung window images. Images were sent to a designated image storage server for later analysis. Following MDCT imaging, the calves were humanely euthanatized with intravenous pentobarbital (Beuthanasia ^®^-D Special, Schering-Plough Animal Health Corp, Union, NJ, USA) at 0.2 ml/kg in accordance with the American Veterinary Medical Association guidelines.

### Image Evaluation

All diagnostic imaging studies were evaluated independently by two evaluators (JF and SSV) using a commercially available DICOM viewer software (eFilm, version 3.3.0, Merge Healthcare, Hartland, WI, USA;) 3D images were created for the figures using a commercially available 3D imaging software (Vitrea workstation, software version 6.3.2, Vital Images Inc., Minnetonka, MN, USA). Each thoracic CR and MDCT study of each calf was evaluated independently and separately by each evaluator. Both evaluators were blinded to the group assignment of the calves and the results of each imaging study. The CR and MDCT studies were evaluated for diagnostic quality and graded as poor, acceptable, and excellent. The imaging studies were graded as excellent if no motion artifacts were present, as acceptable if motion was present to a degree that did not hinder evaluation of the lung parenchyma and pleural surface, or as poor if the study had to be repeated due to extensive motion causing a non-diagnostic study.

Additionally, on CT images the maximum height and length of the right and left lung were measured as well as the maximum tracheal diameter in the mid thorax and at the level just cranial of the tracheal bifurcation. Furthermore, the attenuation of the lung parenchyma was measured in pre- and post-contrast agent injection images at the ventral, mid, and dorsal levels by drawing a circular region of interest (ROI) of 0.3 cm in diameter encompassing lung parenchyma at each level and intercostal space to measure the average Hounsfield units (HU) in this drawn area. Pre- and post-contrast agent injection images were lined up with each other, so that measurements were made in the same anatomic area. No larger vessels or bronchi were included in the ROIs.

**Figure 1:**
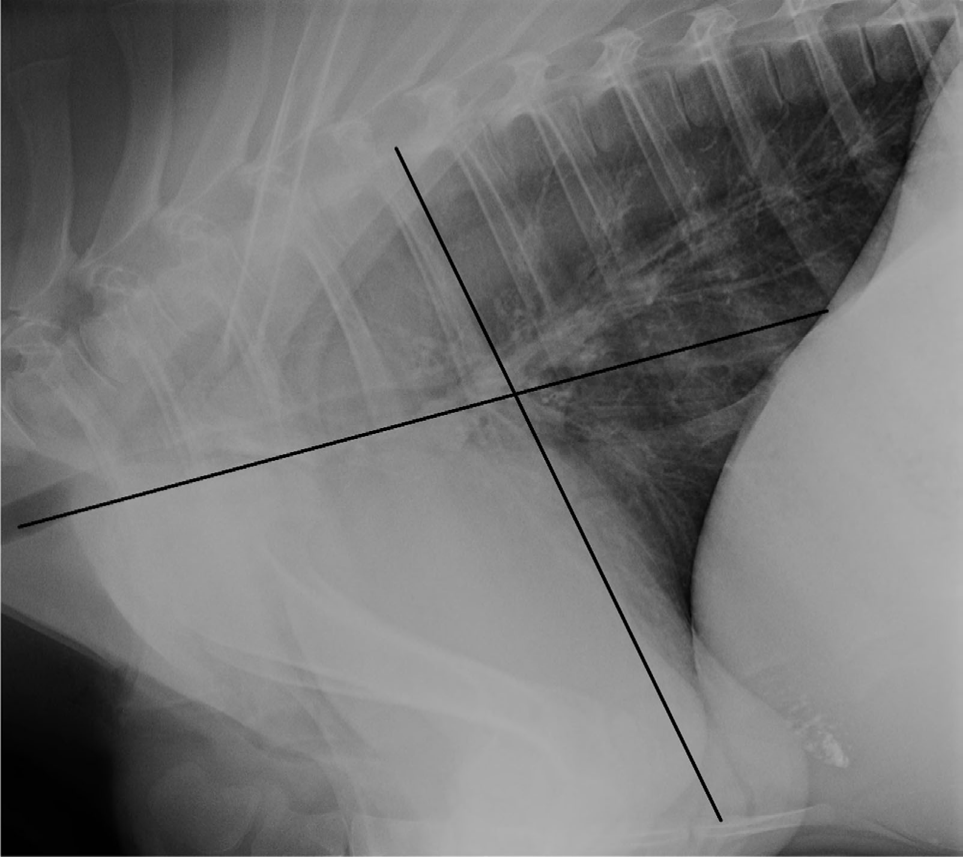
Division of the thorax into four quadrants for pulmonary pattern and severity identification is illustrated on a lateral radiograph of the thorax of a 69-day-old Jersey heifer calf. The line parallel with the ventral aspect of the trachea divides the thorax into a dorsal and ventral half. The line parallel with the caudal border of the 5th rib divides the thorax into a cranial and caudal half.

For evaluation of the lung in each imaging modality, the thorax was divided in four quadrants (Figure 1). The quadrants were defined by a horizontal line running parallel to and at the level of the ventral tracheal margin for a dorsal–ventral delineation, and by a vertical line at the level of and parallel to the caudal margin of the fifth rib for a cranial–caudal delineation. The resulting quadrants of the thorax were craniodorsal (CrD), cranioventral (CrV), caudodorsal (CdD), and caudoventral (CdV). Within each quadrant, alterations of the lung parenchyma and airways were assessed by describing the predominant abnormal pulmonary pattern using the following criteria: presence of prominent and/or thickened bronchial walls (bronchial), a diffuse increased opacity of the lung parenchyma causing loss of definition of the vascular structures (unstructured interstitial), a soft tissue opacity of the lung with the presence of air bronchograms (alveolar with air bronchograms) completely obscuring identification of the vascular structures, borders of the heart or diaphragm, a focal soft tissue opacity in the lung without the presence of air bronchograms (alveolar without air bronchograms), which is completely obscuring identification of other structures. The presence of one or more soft tissue opacity structures measuring up to 3 cm in diameter (nodular), and no abnormality of the pulmonary parenchyma was noted (normal).

In each quadrant of the thorax, an estimate of the percentage of abnormal lung parenchyma was made based on the presence of any pathologic pattern being identified regardless of severity. For example, if 50% of a region was abnormal due to an unstructured interstitial pattern and 50% due to an alveolar pattern with air bronchograms, then this region would be listed as 100% abnormal. If an entire region of lung was considered normal, the region was labeled as 0% to indicate absence of disease.

Furthermore, the presence of pleural fluid was recorded as a yes/no result. The degree of pleural fluid was noted as: within the limits of the ventral third of the thorax (mild), within the limits of the mid third of the thorax (moderate), and extending to the dorsal third of the thorax (severe).

Additionally, the presence of visceral and parietal pleural thickening was recorded as a yes/no answer. The degree of pleural thickening was graded as: less than three focal areas of thickening noted (mild), between 4 and 6 areas of focal pleural thickening noted (moderate), and more than 7 areas of focal pleural thickening noted (severe).

### Pathology and Microbiology

Prior to gross necropsy, nasopharyngeal swabs were collected in each calf for *Mycoplasma* culture and speciation, and *M. bovis* polymerase chain reaction (PCR). During necropsy, lung tissue was collected from various lung regions and submitted for *Mycoplasma* culture and speciation, *M. bovis* PCR, and aerobic bacterial culture and sensitivity. Bacteriology and molecular biology diagnostic evaluations were performed at the Oregon State Veterinary Diagnostic Laboratory (Corvallis, OR, USA). *Mycoplasma* speciation was performed by fluorescent antibody microscopy at the Wisconsin Veterinary Diagnostic Laboratory (Madison, WI, USA). Samples of all major organ tissues were harvested for histopathology.

### Statistical Analysis

Statistical analysis was performed with commercial software (GraphPad Prism, version 6.04, GraphPad Software Inc., La Jolla, CA, USA). Quantitative data were assessed for normality using the Kolmogorov–Smirnov normality test and reported as mean ± SD when normally distributed and as median and range when not. For comparisons between disease groups, quantitative, normal distributed data were analyzed using a one-way ANOVA with Sidak’s multiple comparisons test adjustment for group differences, not normal-distributed quantitative data were analyzed using a Wilcoxon rank sum test, and binary data were analyzed using Fisher’s exact test.

For comparisons of imaging modalities within calf, comparisons of lung quarters within the same calf, and for comparison of evaluators within the same calf, multinomial categorical data were analyzed using a Wilcoxon matched pairs signed rank test and binary paired data were analyzed using McNemar’s test. Categorical data with more than two categories (i.e., disease stage) were collapsed to binary data, if one category dominated the other categories. Inter- and intra-observer agreements were calculated of multinomial categorical data and statistically analyzed using kappa statistics. Statistical significance was set at *p* < 0.05.

## Results

### Animals

Calves in the first-time diagnosis group (group 1) and the short-term chronic group (group 2) were similar in age (40.7 ± 16.2 versus 47.3 ± 13.9 days) and respiratory scores (group 1—9.6 ± 1.5, range: 7–12; group 2—8.3 ± 1.6, range 5–11). Calves in the long-term chronic group (group 3) were older than calves in group 1 and 2 (63.4 ± 19.3 days) and had statistically lower respiratory scores than group 1 (7.6 ± 1.8, range: 4–11; *p* = 0.03). Of the respiratory score criteria, only body temperature differed between groups 1 and 3 (group 1—40.1 ± 0.4 C, group 3—38.7 ± 0.7 C; *p* < 0.0001). All group 1 calves had a body temperature of 39.5 C or greater, whereas only 3 calves in group 2 and 1 calf in group 3 had a body temperature of 39.5 C or greater.

All 30 calves selected for inclusion in the study survived the imaging procedures without the need for additional medical care or sedation reversal agents.

### Thoracic Computed Radiography (CR)

All, but one radiographic study was performed with the calves standing. One calf was unwilling to stand, so the calf was positioned sternally with the forelimbs extended cranially and left-to-right lateral radiographs of the thorax were obtained. Two radiographs of the thorax were sufficient in all calves to ensure that the entire thorax was included within image collimation. All radiographic images were of acceptable to excellent quality and no repeat radiographic studies were required.

### Thoracic Multi-Detector Computed Tomography (MDCT)

Pre-contrast studies were obtained in 100% of patients. Of the survey scans, all were deemed suitable for evaluation by both evaluators, without a marked degree of motion artifact to cause inhibition of pulmonary parenchyma evaluation.

Of the pre-contrast studies, 8/30 (26.7%) cases had negligible motion artifact, 17/30 (56.7%) cases had a mild amount of motion artifact causing mild loss of distinction of the margins of the tertiary bronchi, and 5/30 (16.6%) cases had a moderate amount of motion artifact causing complete loss of distinction of the tertiary bronchi and a mild loss of definition of the larger secondary bronchi. No pre-contrast studies had marked motion artifact.

Intravenous contrast medium administration failed for one case, in which the jugular vein catheter displaced when moving the animal and the contrast medium leaked into the perivascular space of the neck. Post-contrast images were successfully obtained in 97% of cases (29/30). Of the 29 cases in which post-contrast images were available for evaluation, all MDCT scans were deemed suitable for evaluation.

All studies had some degree of respiratory motion induced slice mismatch between the pre- and post-contrast medium administration acquisitions. All post-contrast medium administration studies had a respiratory motion artifact, with 21/30 (70%) having mild motion, and 8/30 (26.7%) having moderate motion artifact. A marked amount of respiratory motion artifact was seen in 1/30 (3.3%); however, the severity of the artifact was not to the degree that it would prevent diagnostic assessment of the images. In the case of extravasation of contrast medium from the jugular vein, the degree of motion artifact was unchanged between the pre- and post-contrast medium administration images.

The cranial border of the cranial lungs was at the level of the first rib in all but one calf, in which the lung extended mildly cranial to the first rib. The caudal border of the lungs ranged from the level of the 11–13th rib (average 12.0 ± 0.7). The length and height of the right and left lungs as well as the tracheal diameter were not significantly different in any of the groups. The differences were less than 1.1 cm in right and left lung height and length, and less than 0.1 cm in the tracheal diameter between the three groups.

The lung parenchymal attenuation averaged throughout all lung lobes before and after intravenous iodinated contrast agent injection was not statistically different between the groups (Table 1). The average of contrast enhancement throughout all lung lobes was not statistically different between the groups and averaged in group 1 = 32.7 HU, in group 2 = 24.6 HU and in group 3 = 30.4 HU. The maximum difference between before and after intravenous iodinated contrast agent injection images averaged 54.3 HU in group 1, 49.3 HU in group 2, and 52.8 HU in group 3. The average lung attenuation in the CrV aspect of the lung lobes (−140 ± 290.7 HU, range −828.0 to 64.3 HU) was higher than in the CdD aspect of the lung lobes (−693.7 ± 121.0 HU, range −851.4 to 26.2 HU).

**Table 1:** Summary of the average ± SD [range] lung attenuation in Hounsfield units (HU) pre- and post-intravenous iodinated contrast medium administration and the difference in attenuation between the pre- and post-intravenous iodinated contrast medium administration images sorted by group.

Disease group	Attenuation (HU) pre-contrast	Attenuation (HU) post-contrast	Difference in attenuation between pre- and post-contrast
Group 1—acute	−456.8 ± 81.9 [−898.9–76.6]	−424.1 ± 82.3 [−871.9–120.4]	32.7 ± 14.6 [11.9–54.3]
Group 2—short-term chronic	−478.2 ± 123.2 [−851.5–123.2]	−451.1 ± 130.3 [–836.2–125.8]	24.5 ± 17.8 [−1.3–49.3]
Group 3—long-term chronic	−521.9 ± 164.1 [−869.1–164.0]	−491.5 ± 165.0 [–841.2–108.1]	30.4 ± 11.2 [16.1–52.9]

### Comparison of Thoracic Computed Radiography (CR) and Computed Tomography (CT) for Pulmonary Disease Diagnosis

**Figure 2:**
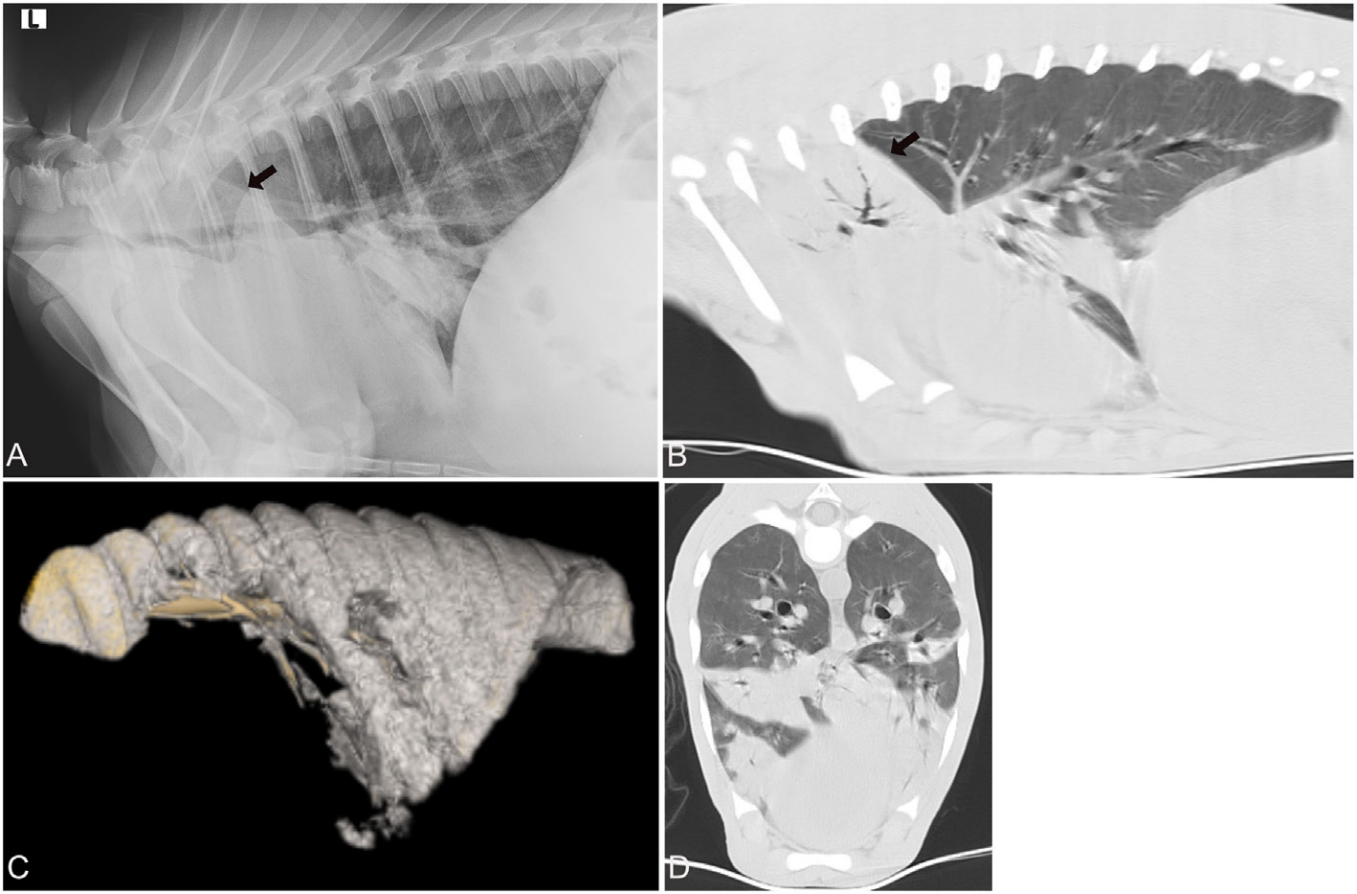
Radiographic and computed tomography images of the thorax of a calf from group 1 (acute respiratory disease) with a respiratory score of 7 and body temperature of 40.5 C. *Mycoplasma bovis* was isolated and a bacterial coinfection was present. **(A)** Lateral radiograph and **(B)** sagittal reconstructed computed tomography (CT) image of the thorax in a lung window illustrating the alveolar lung pattern (*black arrow*) involving both cranial lung lobes, especially the right cranial lung lobe. **(C)** 3D reconstructed image of the air-filled lung. The cranial and cranioventral aspects of the lungs lack air-filling and are therefore not 3D reconstructed. **(D)** Transverse image of the thorax at the caudal aspect of the cardiac silhouette demonstrates the various regions in the lung with an alveolar lung pattern.

**Figure 3:**
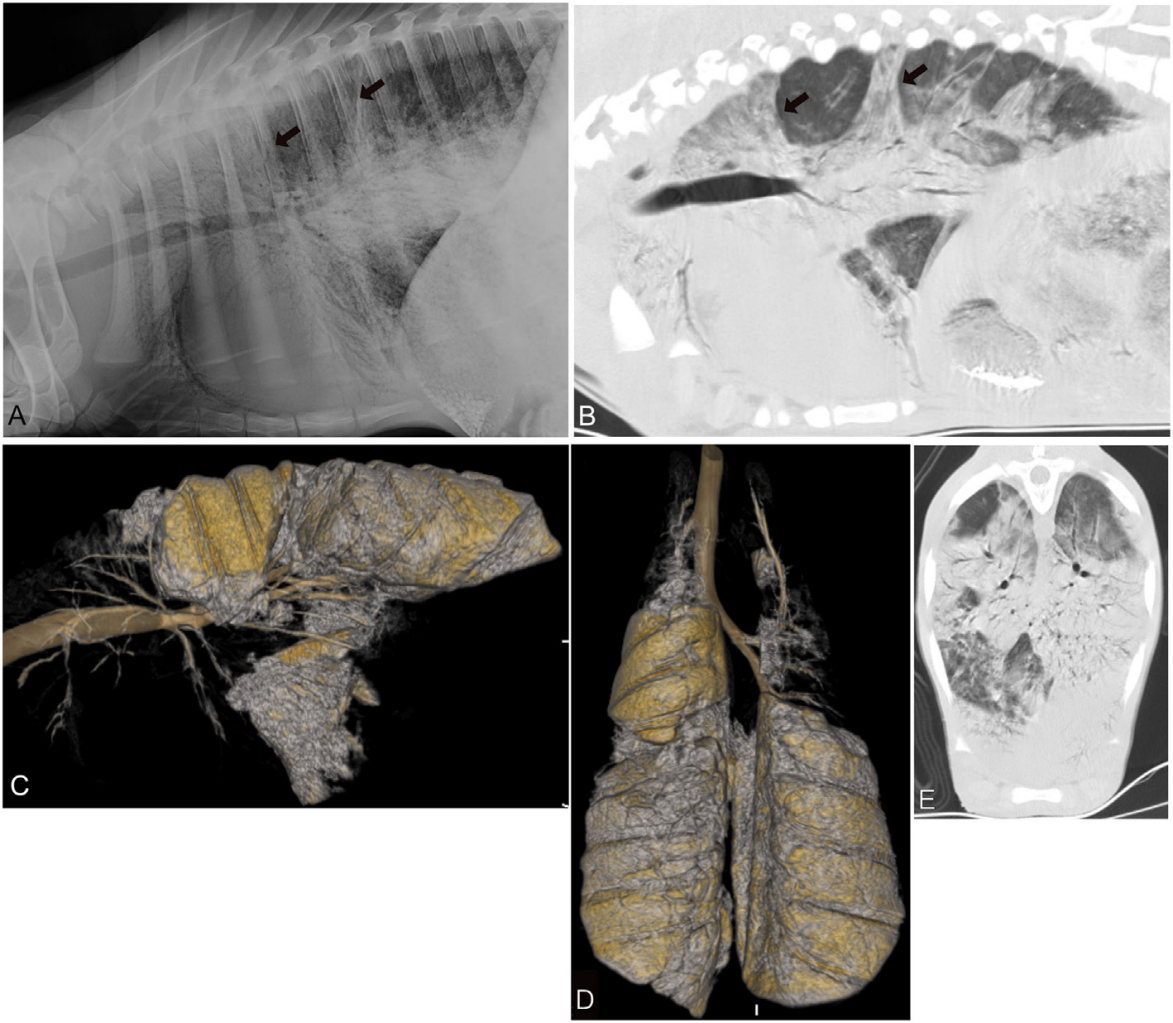
Radiographic and computed tomography images of the thorax of a calf from group 3 (chronic-long term respiratory disease) with a respiratory score of 9 and body temperature of 39.1 C. **(A)** Lateral radiograph and **(B)** sagittal reconstructed computed tomography image of the thorax in a lung window illustrating the various areas of alveolar lung pattern (*black arrow*) involving the cranial aspects of the thorax most severely and to a lesser extent the caudodorsal aspects of the lungs. Three-dimensional (3D) reconstructed sagittal **(C)** and dorsal **(D)** image of the air-filled lung. The entire cranial and in part caudoventral aspects of the lungs lack air-filling and are therefore not 3D reconstructed. **(E)** Transverse image of the caudal thorax illustrating the various areas with a severe lung pattern occupying nearly all aspects of the lung parenchyma.

**Figure 4:**
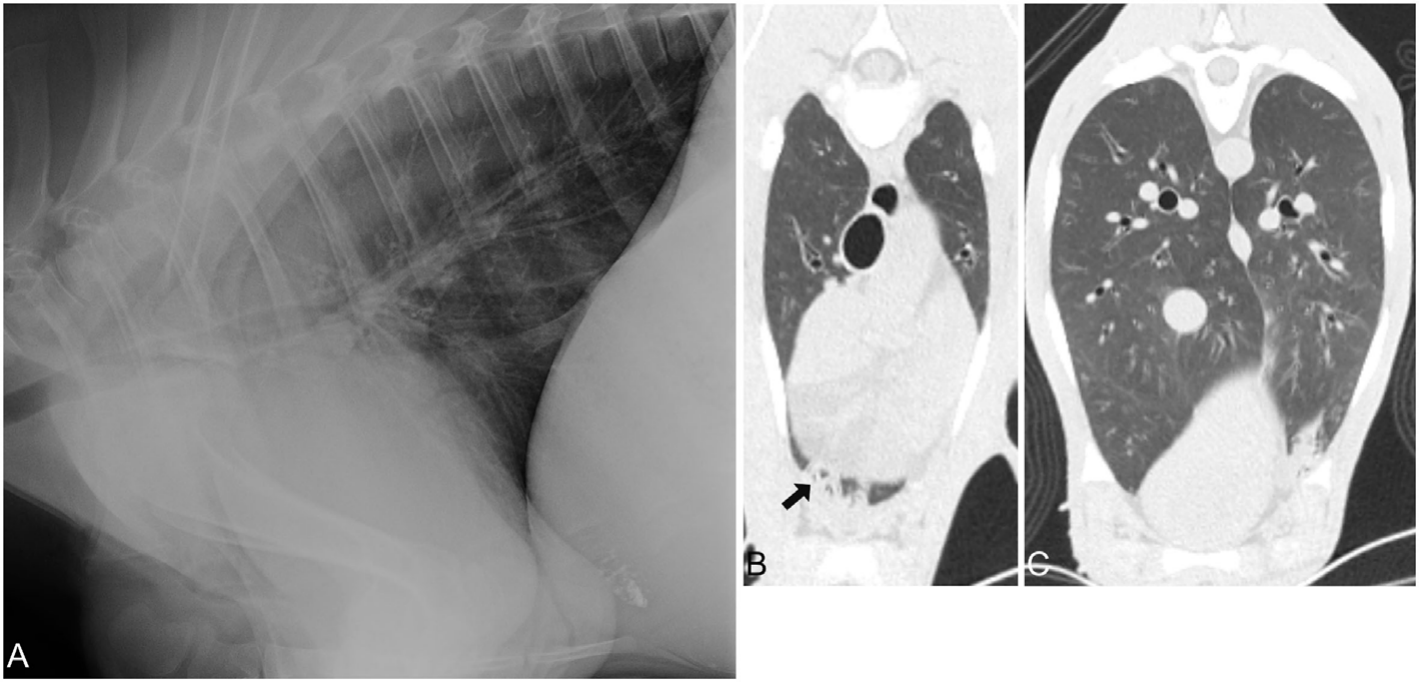
Radiographic and computed tomography images of the thorax of a calf from group 3 (chronic-long term respiratory disease) with a respiratory score of 7 and body temperature of 38.1 C. No bacteria or *Mycoplasma bovis* were isolated. **(A)** Lateral radiograph of the thorax demonstrating minimal lung parenchymal changes of the most ventral and cranial aspects of the lungs. Most aspects of the lung are normally air filled. Transverse CT image of the cranial **(B,C)** caudal aspect of the lungs illustrating that only minimal alveolar changes are noted in the cranioventral aspects of the lungs, and no pathology was noted caudoventral and -dorsal.

Both evaluators detected abnormal lung patterns, consistent with pulmonary disease, in all 30 calves with CT. Using the CT diagnosis of the more experienced evaluator as gold standard, the more experienced evaluator (SSV) also detected abnormal lung patterns in all 30 calves with CR (Figures 2–4). This was repeated when only the CrV quadrant was assessed, in which all patients had disease identified on both CT and CR. Using CR, the less experienced evaluator (JF) correctly identified 27 of 30 calves with abnormal lung patterns, and the three misidentified calves were chronic disease groups (two short-term and one long-term). Differences in abnormal lung detection between imaging modalities were not statistically significant (*p* = 0.25).

The severity of pulmonary disease diagnosis was evaluated based on lung pattern, for which alveolar was considered the most severe pulmonary disease pattern. The more experienced evaluator detected alveolar lung patterns in 29 calves using CT and correctly identified 28 of these calves as having an alveolar lung pattern and 1 calf as not (*n* = 1) using CR. The less experienced evaluator identified correctly 29 of 30 calves having either an alveolar lung pattern (*n* = 28) or not (*n* = 1) with CT and identified correctly 25 of these calves as having an alveolar lung pattern with CR. Four of the misdiagnosed calves were from the chronic disease groups (two short-term and two long-term), and one calf was from the acute disease group. Differences in severity assessment between imaging modalities were not statistically significant (*p* = 0.25).

Regardless of group, all of the calves had at least one lung quadrant with abnormal pulmonary patterns identified; none of the calves had a normal evaluation of all four quadrants of the lungs (Figure 2). In most calves (23 of 30 cases: group 1—nine calves, group 2—seven calves, group 3—seven calves) all four quadrants had an abnormal lung pattern (Table 2). Four calves (group 1—one calf, group 2—one calf, group 3—three calves) had three abnormal lung quadrants. Three calves (group 2—one calf, group 3—one calf) had two abnormal lung quadrants. The CrD and CrV quadrants had abnormal lung pattern in all calves (Figures 2–4). Of the two cranial quadrants, the ventral part was more severely affected, as alveolar lung patterns were observed only in the CrV but not in the CrD quadrant in 8 out of 30 calves (*p* = 0.01) by one evaluator (SSV) and 10 out of 30 calves (*p* = 0.009) by the other evaluator (JF). The three calves with two abnormal lung quadrants had normal CdV and CdD lung quadrants, whereas the four calves with three abnormal lung quadrants had normal CdD lung quadrants.

**Table 2:** Summary of the lung pattern evaluation of each evaluator by modality (CR and CT) in each quadrant of the thorax.

	Modality	CR-CdD						CR-CdV						CR-CrD						Cr-CrV								Total	
Lung pattern		Alveolar		Interstitial		Normal		Alveolar		Interstitial		Normal		Alveolar		Interstitial		Normal		Alveolar		Interstitial		Bronchial		Normal			
Evaluator		Ev2	Ev1	Ev2	Ev1	Ev2	Ev1	Ev2	Ev1	Ev2	Ev1	Ev2	Ev1	Ev2	Ev1	Ev2	Ev1	Ev2	Ev1	Ev2	Ev1	Ev2	Ev1	Ev2	Ev1	Ev2	Ev1	Ev2	Ev1
Normal	CT-CrV																											0	0
Interstitial																				2						1		3	0
Alveolar																				23	29	1	1	1		2		27	30

Normal	CT-CrD													1		2												3	0
Interstitial														2														2	0
Alveolar														17	21	4	8	4	1									25	30

Normal	CT-CdV																											0	0
Interstitial								2				4	2															6	2
Alveolar								15	20	7	8	2																24	28

Normal	CT-CdD																											0	0
Interstitial		1					7																					4	7
Alveolar		20	11	5	9	1	3																					26	23

Total		21	11	5	9	1	10	17	20	7	8	6	2	20	21	6	8	4	1	25	29	1	1	1	0	3	0	120	120

*CR, computed radiology; CT, computed tomography; CrV, cranioventral; CrD, craniodorsal; CdV, caudoventral; CdD, caudodorsal*.

The less experienced evaluator (JF) was less likely to identify disease in the CrD quadrant than the more experienced evaluator (SSV) [80 versus 100% (*p* = 0.01)]. Similar trends were observed for the CrV quadrant (90 versus 100%) and the CdV quadrant (77 versus 90%). No clear trends were observed for the CdD quadrant, as five calves were identified as abnormal only by SSV and six calves were identified as abnormal only by JF. No group differences were detected, when comparing the acute with the chronically diseased calves.

Both evaluators had a moderate to high agreement between CR and CT for identifying diseased lung and lung pattern in the CrV quadrant with a Kappa of 0.70 and 0.96, respectively. Similarly, the inter-observer agreement for CR showed a high correlation with 0.97 and a moderate for CT with 0.70 for the CrV lung aspects (Table 3). All other areas had a lower inter-observer correlation. The least agreement between the two modalities was noted in the dorsal and caudal aspects of the lung (Table 4). Both evaluators identified the CrV quadrant as the most severely and extensively affected quadrant in all three groups. Alveolar pattern was frequently detected in the CrV quadrant on both CT (97%) and CR (93%), and the CrV quadrant had the highest area of alveolar pattern detected on both modalities (CT = 95%, CR = 93%). This was followed by the CrD and CdV quadrants of the thorax. Of these two quadrants, the CdV was more extensively affected compared to the CrD quadrant. The least affected quadrant in all groups was the CdD quadrant using CR in which 10% of calves had an alveolar pattern, and this was similarly reported by both evaluators.

**Table 3:** Intra-observer agreement between radiography (CR) and computed tomography (CT) identifying a normal and abnormal lung patterns in the four quadrants of the thorax.

Quadrant	CrV		CrD		CdV		CdD		Total	
Evaluator	Ev1	Ev2	Ev1	Ev2	Ev1	Ev2	Ev1	Ev2	Ev1	Ev2
Agreement	29	23	21	17	20	15	11	10	81	75
By chance	1	7	9	13	10	15	19	20	39	45
Kappa	0.97	0.70	0.57	0.24	0.50	0	−0.73	0.5	0.52	0.4

*CrV, cranioventral; CrD, craniodorsal; CdV, caudoventral; CdD, caudodorsal*.

**Table 4:** Inter-observer agreement for radiography (CR) and computed tomography (CT) identifying a normal and abnormal lung patterns in the four quadrants of the thorax.

Modality/quadrant	CR-CrV	CR-CrD	CR-CdV	CR-CdD	CT-CrV	CT-CrD	CT-CdV	CT-CdD	Total CR	Total CT
Agreement	29	21	20	11	23	17	15	20	87	69
By chance	1	9	10	19	7	13	15	10	33	51
Kappa	0.97	0.57	0.50	−0.73	0.70	0.24	0	0.50	0.62	0.26

*CR, computed radiography; CT, computed tomography; CrV, cranioventral; CrD, craniodorsal; CdV, caudoventral; CdD, caudodorsal*.

Generally, no significant differences were seen in the extent of affected lung between the three groups. The caudal quadrants tended to have a larger area of lung involved with longer disease duration. Otherwise, no strong correlation was noted between the lung pattern and length of disease course, or extent of diseased lung and length of disease course. In the earlier phase of disease (group 1) both evaluators had a better agreement in diagnosing lung pattern and extent of diseased lung. The largest disagreement between the evaluators was noted in the CrD, CdD, and CdV quadrants of the thorax in regards to lung pattern present. However, both evaluators were in close agreement that the lungs were extensively diseased.

No pleural fluid was noted in either imaging modality. A slightly higher number of cases with pleural thickening were noted using CT than CR (83% using CT, compared to 76% using CR). The degree of pleural thickening ranged from mild to moderate and decreased mildly with chronicity of disease in all calves. In none of the cases, a diffuse or focal contrast enhancement of the pleura was noted. No statistically significant difference was noted between the acute and chronic diseases calves and between the individual groups.

No strong correlation was identified between the CR and CT imaging findings and the respiratory scores.

### Histopathology and Microbiology

Similar to the imaging findings, all calves were histopathologically diagnosed with suppurative pneumonia (Table 5). Two calves (one each in group 1 and 2) were histopathologically additionally diagnosed with necrotizing pneumonia. The CrV aspects of the lungs were the most commonly and severely diseased areas of the lungs. The more dorsal lung aspects were either normal or had only mild changes suggestive of pneumonia. One calf in group 3 had mostly normal lung with only small areas of minimal consolidation. In one calf (group 1), histopathology and microbiology results of the lung tissue were unavailable.

**Table 5:** Summary table of the histopathology, *Mycoplasma* speciation, and bacteriology results including body temperature and respiratory scores sorted by groups.

Disease group	Suppurative pneumonia	Necrotizing pneumonia	*Mycoplasma* in lung total	*M. bovis* in lung	Other *Mycoplasma* in lung	*Pasteurella multocida*	*Mannheimia haemolytica*	Other bacteria lung	Body temperature (C)	Respiratory score

									Average ± SD [range]	Average ± SD [range]
Group 1—acute	10	1	8	6	*M. bovirhinitis* (1), *M. bovigenitalium* (4)	4	3	Mixed gram-positive bacteria (2), *Trueperella pyogenes* (1)	40.1 ± 0.5 [39.5–40.9]	9.6 ± 1.5 [7–12]

Group 2—short-term chronic	10	1	7	6	*M. bovirhinitis* (1), *M. bovigenitalium* (1)	4	1	Mixed gram-positive bacteria (4), mixed gram-negative bacteria (2), *Corynebacterium* sp. (1)	39.3 ± 0.5 [38.5–39.9]	8.3 ± 1.6 [5–11]

Group 3—long-term chronic	10		8	7	*M. akalescens* (2)	7	2	Mixed gram-positive bacteria (2), *Bacillus* sp. (1), α-hemolytic *Streptococcus* sp (1)	38.7 ± 0.8 [37.6–39.5]	7.6 ± 1.8 [4–11]
Total	30	2	23	19	0	15	6			

*Group 1—acute respiratory disease; group 2—short-term chronic respiratory disease; group 3—long-term chronic respiratory disease*.

Twenty-three calves (group 1—eight calves, group 2—seven calves, group 3—eight calves) had *Mycoplasma* isolated from the lung tissue (Table 5). *Mycoplasma bovis* was found in all but three positive cultures, in these three cultures *M. bovirhinis* was isolated. Additionally, in some calves other *Mycoplasma* species, including *M. bovigenitalium* (three calves) and *M. alkalescens* (three calves), were isolated. *Pasteurella* spp. was cultured from the lung tissue of 15 calves and all of these calves had co-infection with *Mycoplasma*. Only one *Mycoplasma* positive calf in group 3 had no bacteria recovered from the lung tissue. Only two calves in group 3 were negative for *Mycoplasma* in the lung tissue and had infections with *Pasteurella multocida*. In only one calf in group 3, in which minimal changes were seen on histopathology, no *Mycoplasma* or bacteria were isolated from the lung tissue.

## Discussion

In our study, radiographs of the calves were obtained in standing position, in all but one calf. All radiographs allowed evaluation of the CrV lung area and diagnosis of the area as diseased despite the summation with the forelimbs. In one study, calves were lifted from the floor to extend the front legs cranially (17), which required at least three persons holding the patient and pulling the legs away from thorax. In our study, usually only one person was holding the standing calf, which minimized the number of people close to the radiographic beam. This also decreased handling of the patient, which might further reduce the stress in the case of a respiratory distressed patient. Although intravenous sedation can be utilized to decrease the patient’s stress and for chemical restraint, all calves enrolled in this study tolerated standing radiographs well while being awake. Furthermore, sedation may result in a decreased respiratory rate and effort and may therefore lead to incomplete aeration of the lung (atelectasis), which can be confused with lung disease such as pneumonia.

The intravenous sedation protocol utilized in this study provided a plane of sedation that was adequate for acquiring MDCT images of diagnostic quality without requiring repeat CT scanning. This is consistent with reports in small animals describing the use of MDCT in awake or sedated animal for the acquisition of pelvic CTs for trauma evaluation in dogs, the abdomen in dogs with acute abdominal signs, and studies of the cat respiratory tract for assessment of upper airway obstruction (11–13, 15). Although calves with respiratory disease commonly have tachypnea and cough as well as other signs of respiratory compromise, the plane of sedation provided by the sedation protocol described herein allowed acquisition of CT images with a generally mild degree of respiratory motion, which did not preclude evaluation of the bronchial tree or pulmonary vasculature or parenchyma. In this study, the intraluminal contents of the bronchi could be evaluated to the level of the tertiary bronchi with ease in almost all cases. Although a degree of blurring of the smaller airways was appreciated in most of the patients, an evaluation of the Hounsfield units in the bronchial lumen could be made to enable identification of a lack of air-filling and abnormal bronchial intraluminal content.

The increased degree of respiratory motion noted in the post-contrast-enhanced MDCT images is likely due to the longer time post administration of sedation, but could in part also be due to the intravenous administration of a low-osmolar non-ionic iodinated contrast agent (iopamidol). It cannot be excluded that some of the calves had an immediate drug reaction leading to an increase in respiratory rate; however, currently no immediate adverse contrast agent reactions are reported in bovine species. For comparison, the percentage of immediate allergic drug reactions reported in human patients post injection of a low-osmolar non-ionic iodinated contrast agent is low ranging between 0.2 and 2.7%. The human allergic drug response most frequently reported post intravenous low-osmolar non-ionic iodinated contrast agent injection included rash (85.3%), itching sensation (58.8%), nausea, and vomiting (6.8%) followed by dyspnea, which was reported in 4.8% of cases in one study (18). No rash was noted in any of the calves post contrast agent injection. However, several of the signs noted as an immediate drug reaction in human patients could not be assessed in these calves. No anaphylactic responses secondary to the iopamidol contrast agent injection were noted in any of the calves in this study. Intravenous contrast medium was administered in this study to assess if the contrast enhancement pattern or degree could be utilized as an indicator of lung disease and disease duration; however, no statistically significant differences were seen in the enhancement between the three groups. Contrast medium enhancement can be utilized to identify if soft tissue attenuating material within the pleural space is due to fluid or vitalized tissue. In this study, no pleural fluid was seen in any patient. Had pleural fluid been present, contrast enhancement might have helped to provide assessment of which pleural layer, parietal or visceral, was thickened. The pleural thickening seen likely involved both pleural layers, but the visceral component was more extensive.

The CrV aspects of the lungs (Figures 2 and 3) were the most commonly and severely affected lung areas in both imaging modalities and on histopathology, which is similar to previous reports (5). The least affected quadrant was the CdD quadrant, and on CR the CdD quadrant had the lowest disease detection rate (60%). As well, there was a higher variability of assessment between the examiners in the CdD quadrant suggesting that this region is more difficult to diagnose as abnormal, especially in the chronic disease cases. Examination of the CdD quadrant alone may be insufficient for detection of diseased patients. This is important to consider when imaging studies are performed as it has been suggested that radiographs of the CdD thorax are more easily obtained likely due to the lack of summation with the soft tissues from the forelimbs, and requiring less high exposure values, when compared to the CrV thorax using radiography (5). Given that radiography is a more accessible and on-farm available imaging modality for veterinarians, this is likely especially important to consider when attempting high quality thoracic radiographs using mobile radiographic units under field conditions where higher radiographic output setting to penetrate the CrV aspect of the thorax might not be available. Furthermore, obtaining radiographs of just the CrV aspect of the lungs might be sufficient to diagnose pneumonia as these areas were diagnosed with disease in more than 93% of the calves. However, none of the calves was diagnosed as completely normal by either of the evaluators using CR when the entire thorax was radiographed, which is consistent with the histopathology reports where all calves were diagnosed with pneumonia. Experience level impacted the assessment of severity of pulmonary disease, as the less experienced evaluator may underscore severity of disease based on imaging findings; this is unlikely to be clinically significant as the calves were still diagnosed with pulmonary disease requiring treatment. No significant difference between imaging modalities was found. Given these findings, CR and CT are likely equally effective in diagnosing acute and chronic pulmonary disease.

No strong correlation was seen between the clinical respiratory scores and the severity of disease on CR and CT. Mismatch between clinical severity and imaging severity is consistent with the concept of a lag between clinical disease and development of imaging abnormalities.

All calves were histopathologically diagnosed with suppurative pneumonia and two calves had additionally a necrotizing pneumonia. Only one calf had minimal lung changes identified histopathologically. Furthermore, abnormal lung patterns identified on imaging correlated well with the histopathology disease diagnosis. This has similarly been suggested for radiography in a previous experimental study (17).

The vast majority of the calves in the study had *Mycoplasma* isolated from lung tissues, and two thirds of those had a co-infection with *Pasturella* spp. This is similar to human studies, in which *Mycoplasma* is known to be a common agent for acute respiratory infection. Furthermore, it is known from human studies that *Mycoplasma* infection may precede and cause subsequent more severe infections with other viruses and bacteria due to immunosuppression and alteration of the normal respiratory flora by the *Mycoplasma* infections (19, 20). Only one *Mycoplasma* positive calf did not have a coinfection with other bacterial pathogens. It is possible that a fastidious organism was present but was not isolated. Future work such as this should include diagnostics with commercially available viral and bacterial bovine respiratory disease PCR assays.

As all the calves in the current study were diagnosed with pneumonia, an accuracy assessment could not be obtained. This limits the study in that we were unable to extrapolate the utility of the tests for a herd assessment tool. If animals without infectious pneumonia have pulmonary changes on imaging that are similar to findings associated with pneumonia, a false positive test result could occur. False positive diagnoses would lead to unnecessary antibiotic therapy, which may increase the risk of development of antibiotic resistant bacteria and treatment costs. We had expected less severe lung parenchymal changes in the calves enrolled in this study and assumed that more lung areas would have a normal lung pattern therefore allowing to better compare the imaging findings in normal and abnormal aspects of the lungs. However, it is important to note that imaging and histopathology findings were in agreement that the caudodorsal aspects of the lungs were the least affected or most normal aspects of the lungs suggesting that both imaging techniques will allow differentiating between normal and abnormal lung parenchyma.

A potential weakness of the study is the low number of evaluators in each group (*n* = 1); which has likely the largest effect when evaluating the differences between the evaluators. However, the comparison between both imaging modalities is likely less affected, especially considering that only minimal differences were noted.

In conclusion, both CR and sedated MDCT were equally effective in diagnosing acute and chronic pulmonary disease. Our results indicate than a less experienced evaluator can detect abnormal lung patterns with CR and MDCT; however, a less experienced evaluator may underscore disease severity. Although acute and chronic pulmonary disease severity can be assessed by both evaluators with both modalities. Furthermore, thoracic MDCT can safely be performed in sedated calves providing images of diagnostic quality. Combing clinical respiratory scores and imaging findings was inadequate for diagnosing chronicity of pneumonia in calves with naturally occurring pneumonia.

## Ethics Statement

The study was approved by the Oregon State University Institutional Animal Care and Use Committee.

## Author Contributions

SS-V and JF made substantial contributions to the design of the study, data acquisition, analysis, and interpretation. JV and KP made substantial contributions to the design of the study, data acquisition, and interpretation. GB made substantial contributions to the design of the study, analysis, and interpretation. All authors agreed to the final version of the manuscript and have contributed to it.

## Conflict of Interest Statement

The authors declare that the research was conducted in the absence of any commercial or financial relationships that could be construed as a potential conflict of interest.

## Abbreviations

CdD, caudodorsal; CdV, caudoventral; CR, computed radiography; CrD, craniodorsal; CrV, cranioventral; CT, computed tomography; HU, Hounsfield units; MDCT, multi-detector computed tomography; ROI, region of interest.
